# The Role of Inflammation and Oxidative Stress in Rheumatic Heart Disease

**DOI:** 10.3390/ijms232415812

**Published:** 2022-12-13

**Authors:** Beata Franczyk, Anna Gluba-Brzózka, Magdalena Rysz-Górzyńska, Jacek Rysz

**Affiliations:** 1 Department of Nephrology, Hypertension and Family Medicine, Medical University of Lodz, 113 Żeromskiego Street, 90-549 Lodz, Poland; 2 Department of Ophthalmology and Visual Rehabilitation, Medical University of Lodz, 113 Żeromskiego Street, 90-549 Lodz, Poland

**Keywords:** rheumatic heart disease, rheumatic fever, valvular disease, inflammation

## Abstract

Rheumatic heart disease (RHD), an acquired valvular disease, remains an important cause of morbidity and mortality in developing countries. This chronic illness starts from untreated streptococcal throat infection, resulting in acute rheumatic fever (ARF) in susceptible individuals. Repeated infections lead to a chronic phase characterized by the damage of heart valves. Inflammation has been found to play important role in the development of this disease. All the studies presented in this review clearly show the involvement of the inflammatory state in the progression of this disease. However, the exact role of cytokines in inflammation sites remains to be examined, since most studies have so far focused on peripheral blood. Such analysis would provide information on inflammatory mechanisms in situ.

## 1. Introduction

Rheumatic heart disease (RHD) is an acquired valvular disease most frequently occurring in developing countries [1]. RHD has been suggested to be a multifactorial illness involving the environment, host and pathogen, all of them determining disease cause and severity [2]. This chronic progressive disease is caused by rheumatic fever (RF) resulting from untreated streptococcal throat infection [3]. Initially, rheumatic heart disease may occur as a complication of acute rheumatic fever (ARF); however, following recurrent episodes of this disease, RHD enters a chronic phase involving the damage of heart valves [4]. In susceptible children, the fever typically occurs after 2–4 weeks from acute tonsillopharyngitis and it leads to the development of an inflammatory state within the heart, joints, nervous system or subcutaneous tissue [5]. In the course of the first ARF episode usually only mild symptoms are observed [6]. According to estimations, 30–45% of children with rheumatic fever suffer from carditis during acute attacks involving myocardial, endocardial and/or pericardial inflammation, while approximately half of them would progress to RHD later in life [3,7]. The infection with group A beta-haemolytic *Streptococci* triggers an autoimmune reaction resulting from antigen mimicry between *Streptococci* M protein epitopes and heart proteins [7]. Auto-reactive antibodies are responsible for the activation of complement proteins, the development of inflammation and subsequent valve damage in susceptible individuals [8]. However, the pathomechanism of progressive valvular damage in RHD remains hypothetical. Ayoub et al. [9] demonstrated that the prolonged presence of streptococcal carbohydrate antibodies triggered autoimmune reactions and progressive heart valve injury. Surgical removal of the occupied valve was found to decrease these antibodies levels. 

Despite being successfully eliminated in developed countries, RHD remains the most prevalent cardiovascular disease in patients below the age of 25 years [4,10]. The global prevalence of RHD is estimated to be approximately 33.4 million cases, with 230,000 cases per year (<0.5 per 100,000 in developed countries and more than 100 per 100,000 in developing countries) and 319,000 deaths annually [11,12]. Corticosteroids and salicylates which are used in acute RF attacks can relieve symptoms; however, such short-term treatments do not alter the long-term outcome of RHD [13]. The current treatment of RHD appears to be primarily focused on the prevention of acute attacks of RF, not on the protection against the progression of valvular damage [7].

## 2. Rheumatic Heart Disease (RHD)

The cardiovascular risk in patients with rheumatic diseases is partly associated with systemic inflammation, and partly with the presence of cardiovascular risk factors, including age, gender, smoking, dyslipidaemia, family history and a sedentary lifestyle [14]. The abundance of inflammatory cells as well as cytokines, chemokines, adhesion receptors, proteases and autoantibodies is vital for vascular dysfunction leading to accelerated atherosclerosis, but it can also affect cardiovascular system structures, including cardiac valves, myocardium, pericardium and the conduction system [14,15]. Cardiac involvement in the course of acute rheumatoid fever is also triggered by the interaction between streptococcal antigens and the valve tissue [8]. However, the mechanisms underlying valve damage in RHD patients are not so well understood. Some studies have demonstrated the presence of mineralization in areas of inflammation and neoangiogenesis with increased expression of vascular endothelial growth factor (VEGF) [16]. It is also plausible that mitral valve mineralization in RHD involves calcification-competent extracellular vesicles originating from smooth muscle cells, VICs or macrophages [17,18,19]. According to hemodynamic theory, progressive valve damage is due to continuous trauma resulting from turbulent flows via a valve that was deformed in the initial stage of rheumatic fever [7]. It is also plausible that valve thickening and deformity are associated with persistent valvular inflammation related to RF (inflammatory theory). Streptococcal antigens, especially M proteins, were found to interact with host cardiac antigens which can lead to the development of carditis and valvulitis [20,21]. The study of a Lewis rat model of rheumatic carditis revealed that M protein-specific T cells acted as key mediators of valvulitis [22]. 

Vascular disorders observed in patients with RHD involve valvular nodules, valvular regurgitation and vegetations [14]. Kanagasingam et al. [23] showed that mitral stenosis, mitral regurgitation and aortic regurgitation are the most frequent forms of valvular malfunction in RHD. Guedes et al. [24] demonstrated that mitral regurgitation was present in 80% of patients with rheumatoid arthritis. This form occurs mostly in the early stages of the disease, while stenosis is reported in advanced disease [5]. Approximately 90% of RHD patients present the involvement of the mitral valve in the form of leaflet thickening and calcification as well as a commissural fusion [25]. Apart from the mitral valve, other valves can also be affected by RHD. Aortic and tricuspid valvular diseases are observed in RHD patients, but less frequently [26]. Valve leaflet thickening, nodules and commissural and fusion calcification are the predominant anatomic abnormalities. Kanagasingam et al. [23] found that the majority of RHD patients in their study had partially or completely fused commissures which subsequently led to significant valvular damage. Moreover, they observed a robust correlation between immobility and sub-valvular thickening and the severity of mitral stenosis. The presence of calcification has been suggested to be triggered by lipids, similar to vascular calcification related to atherosclerosis. In the early stages of rheumatic disease, small verrucous nodules can be observed in echocardiographic exams. These nodules are associated with thrombi spreading along the lines of heart valve closure; however, they do not impair valve function and do not result in leaflet damage [6]. The development of valve dysfunction in genetically predisposed patients is the effect of long-term inflammation associated with episodes of rheumatic fever. Pathomorphological examinations have revealed thick and stiff mitral valves resulting from advanced fibrosis in patients with end-stage disease. Moreover, in this group of patients, the fusion of valve commissures, as well as the erosion of endothelium surface, are found. In addition, the fusion and shortening of chordae tendineae could decrease subvalvular chordal space [27,28]. However, this image differs at various disease stages. In young patients, leaflet fibrosis is sparse. Various morphological images translate into different clinical manifestations. In the majority of patients with mitral stenosis, chordal shortening is reported. In turn, this symptom is observed only in 3% of individuals with pure mitral regurgitation. Annular dilatation is highly prevalent (90%) in patients with pure mitral regurgitation but in less than one-third of patients with pure stenosis [6]. Inflammation, as well as hemodynamic injury to valve leaflets, is primarily responsible for the steady progression of RHD. One study has indicated a high percentage of RHD patients with no previous history of acute rheumatic fever (40%) which suggests that they had probably clinically silent acute episodes; however, other data in the literature demonstrate a much lower incidence of mild or asymptomatic ARF [23]. Substantial valvular damage in the course of chronic RHD may require valve intervention or even valve replacement [23]. 

Patients with RHD, due to the persistent inflammatory activity within the atrial myocardium, may also face an increased risk of atrial fibrillation [29,30]. Some patients were observed to have extraordinarily enlarged left atrium (LA) [31]. Apart from a greater haemodynamic burden on the LA, prolonged autoimmune-related inflammation could also favor the LA remodelling in RHD patients. The progressive cardiac valve dysfunction in the course of RHD may be accompanied by arrhythmia and heart failure, as well as thromboembolism [32,33,34]. Due to the fact that not every patient with severe rheumatic mitral stenosis accompanied by substantial hemodynamic stress develops atrial fibrillation, it has been suggested that chronic inflammation may be the characteristic and triggering factor of AF in this group of patients [35]. The authors suggested that chronic inflammation and hemodynamic stress may act synergistically to promote the development and worsening of atrial fibrillation. 

**Figure 1:**
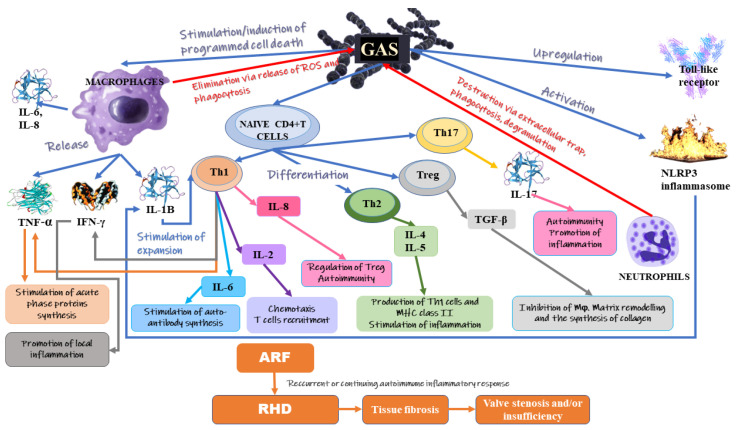
The mechanisms involved in the development of RHD.

The results of studies have identified various factors, including the host’s immune response and genetic predisposition, which affect the progression rate of valvular lesions [36,37]. Some studies have revealed that an enhanced risk of RHD may be associated with high mannose-binding lectin (MBL) levels. Moreover, genotypes related to the high synthesis of MBL stimulate the development of acute and chronic rheumatic carditis [38,39]. MBL has been suggested to trigger inflammation and complement tissue damage, even in patients in the chronic stage of the disease. It appears that MAPK kinase and its key downstream pathways (ERK1/2, p38 kinases, JNKs) are involved in the pathogenesis of RHD [34]. The activation of these pathways is associated, among others, with differentiation, proliferation and the remodelling of ECM, as well as fibrosis (in cases of uncontrolled activity) [34,40,41]. Figure 1 presents the mechanisms involved in the development of RHD.

## 3. Inflammation

Autoimmune rheumatic diseases are characterized by the presence of systemic inflammation [42]. RHD development is associated with cellular and humoral immune responses related to host auto-reactivity [43]. The presence of inflammation and oxidative stress has been suggested to be involved in the pathogenesis of rheumatic heart valve disease [44,45]. Higher levels of soluble factors in patients with active RHD compared to those with latent states indicate greater activation of the immune system in the first group. The infection with group A streptococcus (GAS) can induce delayed autoimmune response which results in ARF, while a recurrent or continuing autoimmune inflammatory response against self-antigens appears to be the trigger involved in the pathogenesis of autoimmune-driven RHD [32,46]. According to studies, a chronic inflammatory reaction occurring in the course of RHD is probably associated with the development of tissue fibrosis, thus leading to valve stenosis and/or insufficiency which may even require valve surgery [29,47]. The mechanisms of pattern recognition of GAS antigens by human immune cells are not fully clear. It has been found that GAS-related M proteins, peptidoglycans and nucleic acids can stimulate the macrophages to release proinflammatory cytokines, including interleukin (IL)-1β and tumour necrosis factor (TNF) [48,49,50]. The activation of antigen-specific cells by antigen-presenting cells is followed by the differentiation of naïve CD4 T cells into effector T helper cell triade (Th1, Th2, Th17), responsible for the shape of the immune response. These cells are accompanied by distinctive cytokine products, profiles of surface receptors and transcription factors [51]. Following infection with GAS, the innate immune response (involving DC, neutrophils and macrophages) is triggered [34]. 

Epithelial cells can also release peptides and cytokines in order to attract immune cell mediators and neutrophil chemotactic factors (IL-8). In addition, the upregulation of Toll-like receptor (TLR) is observed at that time [52,53]. Attracted neutrophils can destroy GAS via neutrophil extracellular trap, as well as phagocytosis and the degranulation of the anti-microbial peptide [54]. Recurrent GAS infections are associated with the higher release of IL-17 from Th-17 which leads to the accumulation of other neutrophils and macrophages [55,56]. In turn, phagocytosis and the release of reactive oxygen species are mechanisms via which resident macrophages eliminate GAS. Moreover, macrophages increase the pool of cytokines since they release IL-6, IL-8, tumour necrosis factor-α (TNF-α) and Interferon-γ (IFN-γ). Subsequent steps lead eventually to the differentiation of CD8+ T cells and B cells which distinguish GAS antigens [57]. The antigenic structure of GAS resembles the human M protein, which in turn is highly similar to the γ-helical coil structure of many valvular proteins, tropomyosin and cardiac myosin [58]. This is the reason for the interaction of T cells with cardiac valves. The formation of autoantibodies triggers a higher synthesis of vascular cell adhesion molecule 1 (VCAM1). VCAM1 upregulation worsens the inflammation by causing the adherence of T cells to the endothelium. Furthermore, the activity of autoreactive T cells is associated with the formation of Aschoff bodies. 

Kim et al. [32] demonstrated that in patients with RHD, the dysregulation of the immune response of peripheral blood mononuclear cells (PBMC) to GAS resulted in the enhanced production of CD4 T cell-derived proinflammatory cytokines (e.g., granulocyte-macrophage colony-stimulating factor (GM-CSF)) which was accompanied by chronically increased IL-1β. Moreover, IL-1β was found to stimulate the expansion of Th1 CD4 T cells that released GM-CSF in a potential feed-forward mechanism. These factors were demonstrated to link adaptive (lymphocyte) and innate (myeloid cells) in autoimmune disorders [59]. Many studies have confirmed that GM-CSFs are involved in the progression of various autoimmune diseases, including autoimmune myocarditis, rheumatoid arthritis, encephalomyelitis and multiple sclerosis [60,61]. Apart from the Th1 CD4 T cells, M1 protein present on the surface of GAS has been demonstrated to induce programmed cell death in macrophages (Mϕ) and stimulate the inflammasome NACHT, LRR and PYD domains-containing protein 3 (NLRP3) [49]. The activation of caspase-1-dependent NLRP3 inflammasome was associated with the maturation and secretion of proinflammatory interleukin-1β (IL-1β) as well as macrophage pyroptosis. 

According to studies, inflammatory infiltrates within the rheumatic mitral valve in end-state disease patients comprise primarily mononuclear cells (CD4, T CD8 lymphocytes, macrophages and B cells) [62,63]. The effector function of these cells is associated with cytokines profile and the soluble mediators produced by them [64]. T CD4+ lymphocytes, which are prevalent in inflammatory infiltrates in RHD, show high cross-reactivity against cardiac myosin epitopes and can differentiate into various subpopulations which release different cytokines [65,66]. The analysis of peripheral blood and valvular tissues collected from RHD patients indicated the preponderance of T CD4+ cells over T CD8+ cells. However, levels of these cells differ between various stages of the disease [62,63]. Inflammatory infiltrates present in rheumatic mitral valves were also found to contain B lymphocytes. These cells were suggested to be involved in the production of antibodies in the early disease stages; however, at more advanced stages of the disease, they may play the role of effector cells contributing to chronic lesion development [63,67]. The inflammation in RHD has been suggested to be associated with the stimulation of T lymphocytes in the presence of macrophages and dendritic cells (DCs) (antigen-presenting cells) [68]. Shiba et al. [29] found higher amounts of infiltrating inflammatory cells, such as T lymphocytes, macrophages and several phenotypes of DCs, in the left atrium of RHD patients compared to the age-, sex- and LA size-matched non-RHD group. This observation implies the constant presence of inflammatory activation in the enlarged LA in RHD. Moreover, it appears that RHD-related remodelling, not haemodynamic-related stress, is associated with LA remodelling in patients with RHD. Recent studies have found that macrophages, which play a deleterious role in cardiovascular diseases, may be also involved in rheumatic diseases. Macrophages (M1) activate the NLRP3 inflammasome resulting in the synthesis of IL-1β and IL-18 [69,70]. The actions of IL-1β are associated with the secretion of matrix metalloproteinases (MMPs) TGF-β and IL-6 leading to proliferation and fibrosis [71]. Enhanced TGF-β expression was found to positively correlate with valvular fibrosis in RHD [72]. High plasma levels of MMP-1 were found to be risk factors for RHD [73,74]. Moreover, the activity of MMPs may be also associated with the regulation of calcification via elastin degradation.

Many studies have confirmed the elevated levels of immunoregulatory cytokines, especially IL-6, TNF -α, IL-10 and IL-4 in patients with severe RHD [75,76,77]. It seems that the tenacity of auto-reactivity in patients with chronic RHD lies behind long-lasting immunological disturbances. In addition, the progression of RHD is associated with the abundance of pro-inflammatory cytokines, including TNF-α, IFN-γ, IL-1, IL-2 and IL-6 [6]. Due to their strong high chemotactic potential, TNF-α stimulates the attraction of cells to the inflammation site. In turn, IFN-γ actions are associated with the promotion of autoantigens processing and presentation [66,78]. Numerous studies pointed to IL-1 as a factor involved in inflammatory damage, particularly in the acute phase of rheumatic disease [79,80]. One of the studies found that polymorphism within IL-Ra and IL-6 genes modulated the susceptibility to RHD [81]. In turn, IL-6 was demonstrated to be involved in B cell antibody production and RVD pathogenesis [82]. Some studies revealed low levels of IL-2 and deficiency of circulating Tregs in patients with rheumatic mitral valve disease [78,83,84]. The first cytokine is involved in the formation of regulatory T cells (Tregs), while Tregs are responsible for the maintenance of immune tolerance. A greater deficiency in the Tregs number was demonstrated to be associated with multiple valve impairment [85]. Some studies have indicated the correlation between high levels of IL-10 (acting as a chemoattractant) and T CD8+ lymphocyte response in RHD patients [77]. 

Several studies have demonstrated patients with chronic RHD altered expression of miRNAs, especially miR-205-3p, miR-1183 and miR-101, which target IL-1β and TLR2 pathways [1,86,87,88]. In addition, the role of miR-155-5p has been suggested in the development of RHD since it participates in inflammation, immunity and fibrosis [89,90,91]. The role of miR-155 in inflammation-induced disorders has been confirmed in many studies. For example, the inhibition of miR-155 was associated with the hampered development of an abdominal aortic aneurysm as a result of diminished macrophage inflammation and the reduction of inflammatory state following myocardial infarction in another study, while the absence of this miRNA translated into decreased inflammation, infarct size and reduced collagen deposition [92,93,94]. Chen et al. [86] demonstrated that the inhibition of miR-155-5p in RHD resulted in reduced inflammation and fibrosis in the valves. Moreover, they suggested that the exosomes may also be involved in valvular damage observed in RHD through the transfer of miR-155-5p. This miRNA was found to directly target S1PR1, a G protein-coupled receptor, via binding to its 3′ UTR in vitro, which resulted in suppressed S1PR1 expression. Furthermore, Chen et al. [86] suggested that miR-155 may regulate the SOCS1/STAT3 signalling pathway in RHD. Based on their findings, the authors concluded that miR-155-5p enhanced the progression of valvular damage via many pathways, while its inhibition could limit this process [86]. The inhibition of miR-155-5p was found to correlate with diminished expression of IL-6 in valves and serum [86]. Another study found that miR-155 stimulates the formation of Th17 cells and Th1 cells subsets [90]. Lu et al. [1] found that hsa-miR-205-3p and hsa-miR-3909 can target the IL-1β-IL-1 receptor pathway, thus enhancing the inflammation level. The abnormal miRNA profile observed in the serum of patients with rheumatic valvular heart disease appeared to enhance the expression of both IL-1β and IL1R1, thus stimulating fibrosis [95]. Lu et al. [1] demonstrated differences in the expression of IL-1β and IL1R1 between patients with rheumatic heart disease and congenital heart disease, which indicated the state of prolonged inflammation despite the lack of streptococcal infection. This phenomenon can stimulate the progression of heart valve stenosis. According to studies, interleukin-1 plays a key role in the development of rheumatic diseases [96]. Interleukin-1 (IL-1α and IL-1β), secreted by monocytes and neutrophils, participates in the activation of innate immunity [1]. IL-1β is a potent proinflammatory cytokine that is responsible for the attraction of leukocytes to the sites of infection, stress and tissue damage [97]. Moreover, it also stimulates matrix enzymes. In vitro exposure to GAS was found to trigger the persistent release of IL-1β which may imply that the inhibitory mechanisms of feedback become dysregulated in patients with RHD [32]. Despite the proven role of the IL-1 family, it remains unclear whether it is also engaged in chronic rheumatic valvular heart disease. Yeğin et al. [79] reported that interleukin-18 stimulated the activation of myofibroblast of valvular interstitial cells. Diamantino Soares et al. [75] demonstrated that patients with severe RHD had higher levels of inflammatory cytokines compared to those with stable diseases. Moreover, they indicated a positive correlation between the expression of IL-6 and TNF-α in individuals with severe RHD and its relationship with severe valve dysfunction [75]. In turn, increased levels of IL-10 and IL-4 were associated with adverse outcomes. The role of IL-4 cytokine is to regulate humoral immune response, promote tissue repair and fibrosis [98]. The analysis of polymorphisms within cytokines genes demonstrated the correlation between the presence of *TNF-α* -308G/A and *interleukin-6* -174G/C SNPs and the susceptibility to RHD. Perhaps the presence of these SNPs is associated with the stimulation of chronic inflammatory state reported in RHD. According to Soares and colleagues [75], the expression of both IL-6 and TNF-α correlated with a worse clinical presentation. 

Bilik et al. [99] reported that markedly higher serum levels of IL-17 and IL-23 in patients with rheumatic mitral valve stenosis (compared to healthy individuals) may play a role in the inflammatory process involved in the development of this disease. Another study found that levels of Th17 cell-related cytokines, such as IL-17 and IL-21, were considerably higher in patients with RHD [100]. In an experimental model, Th17 cells which release IL-17 have been found to be associated with disease progression toward the chronic state [100]. Patients with rheumatic mitral valve disease have elevated amounts of Th17 cells, as well as IL-17 serum levels, compared to healthy individuals [84]. Ali et al. [101] observed higher concentrations of TNF-α as well as normal levels of IFN-γ and IL-10 in RHD patients. Tormin et al. [43] reported increased serum concentrations of soluble factors, such as C-C Motif Chemokine Ligand 5 (CCL5), C-X-C Motif Chemokine Ligand 8 (CXCL8), Interleukin 1 receptor antagonist (IL-1ra), IL-4, IL-9 and platelet-derived growth factor (PDGF), in patients with late RHD. These factors were demonstrated to differentiate between clinical RHD and latent disease with 100% sensitivity and specificity. In turn, serum concentrations of granulocyte colony stimulating factor (GCSF), CXCL8, IL-4, IL-7, IL-15, IL-1ra predicted clinical disease with 100% sensitivity and specificity when compared to healthy controls [43]. Not only the level, but also the occurrence, of some SNPs was associated with clinical disease. Specific *IL6*, *IL10*, *IL2* and *IL4* genotypes also enabled the prediction of clinical RHD. According to studies, the presence of the C allele (*IL4* rs2243250 polymorphism) translates into decreased transcriptional activity and it is much more frequent in patients with clinical RHD [102]. Tormin et al. [43] have revealed a robust correlation between fibroblast growth factor 2 (FGF-2) and PDGF. Since these molecules have pro-fibrotic properties, the authors suggested that they can be involved in valve fibrosis in RHD. Moreover, they observed a relationship between plasma VEGF levels and GM-CSF and implied that these two factors may be involved in the maintaining of the inflammatory state as well as the calcification of valves. 

In addition, increased levels of C-reactive protein (CRP) in RHD patients have been demonstrated in many studies. CRP levels in RHD patients who underwent percutaneous transmitral commissurotomy were significantly decreased which may imply that turbulent flow resulting from damaged valve favors the development of persistent subclinical inflammation and accelerated fibrosis, thickening and calcification [23]. The levels of high-sensitivity (hs)-CRP were found to be markedly higher in those RHD patients who show multi-valvular involvement [103]. Attar et al. [7] demonstrated significantly increased mean plasma hs-CRP levels in patients with rheumatic valve disease compared to the control group, which may support the thesis of chronic inflammation within cardiac valves. Patients with more enhanced inflammatory reactions displayed a more accelerated progression of valvular dysfunction [7]. Moreover, the authors revealed that patients with RHD and higher levels of hsCRP were more prone to atrial fibrillation (AF), left atrial thrombus formation and had an increased risk of metabolic syndrome complications [7,104,105]. One hypothesis stated that CRP can bind to atrial myocyte which results in the initiation of complement-mediated local inflammation and leads, in consequence, to the onset of atrial fibrillation [106]. The measurement of pentraxin-3 (PTX3) levels as an indicator of inflammatory state revealed its higher levels in RHD patients [107]. This study also demonstrated that PTX3 level more accurately correlated with the severity of mitral valve stenosis than hsCRP. The presence of the chronic systemic inflammatory state in RHD patients was also confirmed by the finding of an increased neutrophil-to-lymphocyte ratio (NLR) [108]. The presence of rheumatic mitral valve stenosis was associated with higher NLR (2.9 (0.6–13.0) vs. 2.1 (0.7–5.8), *p* < 0.001) and C-reactive protein (5.99 (0.3–23.7) vs. 2.98 (0.6–6.3), *p* = 0.001) levels compared to healthy individuals [108]. Akboğa et al. [108] also demonstrated that NLR, CRP and left atrial diameter were independent predictors of rheumatic mitral valve stenosis. Another case-control study found a greater mean NLR count in patients with severe multi-valvular RHD than in those with isolated mitral stenosis, mitral regurgitation or both [109]. Increased levels of pentraxin-3 (PTX3), homocysteine and an increased neutrophil-to-lymphocyte ratio have been reported in patients with RHD [108,110,111,112]. Rastogi et al. [2] found increased expression of intercellular adhesion molecule 1 (ICAM1), vascular cell adhesion molecule 1 (VCAM1) and E-selectin in endothelial cells as well as hypermethylation of the ICAM1 promoter which increased ICAM1 mRNA levels in RHD patients. Enhanced expression of ICAM-1 correlated with valvular lesions, death of myocytes and heart failure in another study [113]. Mitral valves from RHD patients also showed considerably increased expression of vimentin (*p* = 0.001) compared to controls. In addition, the promoter of vimentin was found to be hypermethylated in the RHD mitral valve [2].

Some studies have demonstrated cross-reactivity between anti-streptococcal antibodies, N-acetyl-β-D-glucosamine (GlcNAc) and myosin present in the sera collected from patients with rheumatic fever [34]. Anti-GlcNAc/anti-myosin was found to be cytotoxic for human endothelium cells cell lines [114]. Moreover, it reacted with both human valvular endothelium and underlying basement membrane which is in agreement with the thesis that these cross-reactive antibodies can damage the endothelium and underlying matrix. The cytotoxicity is associated with inflammation. In addition, considerable upregulation of sST2, which is reported in patients with RHD, promotes continuous inflammation, thus aggravating valvular damage in the course of RHD [115]. In turn, the activation of JNK was found to enhance the proliferation of fibroblast and collagen deposition in the ECM of cardiac valves. All these mechanisms are responsible for both calcification and stiffening of the heart valves in RHD [116]. 

The most recent studies have hypothesized that RHD is associated with collagen autoimmunity rather than with molecular mimicry or impairment of the immune system [117]. Following Streptococci infection, M protein attaches to the CB3 region of collagen IV forming a complex that induces conformational changes in the collagen structure, thus triggering an anti-collagen response [118,119]. Therefore, a pervasive protein can act as a self-antigen which results in the disparities between collagen deposition and degradation. Such a sequence leads to fibrosis of the valves in RHD [6]. The analysis of mitral valves indeed demonstrated increased deposition of collagen Type I and Type III and fibrosis compared to the control group without rheumatic mitral valve disease [73]. 

## 4. Oxidative Stress

Levels of antioxidants and reactive oxygen species formed as products of oxygen metabolism are in balance in our body [120]. However, some pathologic conditions are related to the shift of balance towards reactive oxygen species (ROS) production, thus creating oxidative stress [121]. ROS affects many signaling pathways involved in cell growth, cellular differentiation, apoptosis, immunity and intracellular messaging, and is responsible for DNA damage, lipid peroxidation and damage of proteins, as well as enzyme oxidation [122,123]. Oxidative stress has been found to promote the development of various diseases, such as atherosclerosis, cardiovascular diseases, cancer, metabolic disorders and diabetes [124,125]. Enhanced oxidative stress was suggested to be involved in the pathogenesis of autoimmune disorders as a result of promoting the inflammation and apoptotic cell death and impairing the immunological tolerance [126]. It also appears that oxidative stress plays a role in RHD; however, the amount of evidence is much sparser compared to that for inflammation. There are a few studies assessing the parameters of oxidative stress in patients with RHD [122,127,128]. Chiu-Braga et al. [128] demonstrated markedly higher levels of advanced oxidation protein products in rheumatic heart valve disease (RHVD) patients compared to controls. However, levels of these products did not correlate with the commissural severity of mitral disease in the study group. In addition, Karatas et al. [127] indicated higher oxidative status in patients with rheumatic heart valve disease compared with a control group. In turn, Rabus et al. [122] analyzed plasma and tissue oxidative stress markers in patients with rheumatic and degenerative HVD. They observed no differences in plasma total oxidant status (TOS), total antioxidant status (TAS) and oxidative stress index (OSI) between these two diseases. In addition, tissue levels of TOS and OSI were similar in both groups. Similarly, Sari et al. [120] failed to observe considerable variances in systemic oxidative state between patients with RHVD compared to control. According to the authors, these discrepancies between studies may be associated with different severity of the disease in studied patients and different measures used to assess parameters in the aforementioned studies [120]. Moreover, the study of pediatric patients with RHVD and congenital heart valve disease did not show differences in serum oxidative and antioxidative parameters between groups, which suggests that oxidative stress may not exert a significant role in the pathogenesis of rheumatic HVD in childhood [127]. As a result of oxidative stress in RHD patients, they experience higher cardiovascular morbidity and mortality compared to the general population [129]. In rheumatic diseases, the presence of an inflammatory state contributes to endothelial dysfunction. The combination of reduced NO bioavailability and enhanced ROS production by NADPH oxidase and eNOS uncoupling promotes the progression of atherosclerosis. Inflammation-related constant production of interferon-alpha (IFN-α) and enhanced expression of IFN-α-regulated genes have been shown to modulate oxidative stress pathways [130]. IFN-α associated depletion of BH4 was found to result in eNOS uncoupling and oxidative stress [131,132]. Results of animal and human studies have confirmed that IFN can accelerate atherosclerosis, while anti-TNF therapy has the potential to ameliorate vascular function, thus decreasing the development of premature atherosclerosis [129,133]. Moreover, peripheral blood monocytes and neutrophils of patients with rheumatic fever (RF) and rheumatic heart disease (RHD) can produce oxygen free radicals (OFR) [134]. The release of OFR is especially high in patients with recurrent rheumatic activity compared to chronic RHD. Kumar et al. suggested that the generation of OFR within the myocardium may contribute to the pathogenesis of cardiac damage observed in patients with RHD [134].

The results of human studies concerning the role of inflammation and oxidative stress in RHD patients are summarized in Table 1.

## 5. Treatment

Recent recommendations in the 2020 Australian Guidelines for Prevention, Diagnosis and Management of Acute Rheumatic Fever and Rheumatic Heart Disease suggest that secondary prophylaxis in patients diagnosed with acute rheumatic fever or rheumatic heart disease can be shortened in some individuals without cardiac involvement [135,136]. Non-steroidal anti-inflammatory drugs (NSAIDs) including ibuprofen or naproxen are considered first-line drugs. These drugs are used in high dose at the beginning; however, after 1–2 weeks the dose can be reduced. Additionally, proton pump inhibitor therapy should be administered together with long-term therapy. Moreover, patients with rheumatic heart disease are recommended endocarditis prophylaxis [136]. Due to its less favorable safety profile, aspirin is used as second line drug.

The management of symptoms in acute rheumatic fever involves the eradication of streptococcal infection (a trigger), alleviation of cardiac damage and mitigation of symptoms, as well as prevention of recurrences [136]. Due to the persistent inflammatory state in RHD, the use of nonsteroidal anti-inflammatory drugs and glucocorticoids is associated with the relief of symptoms; however, such treatment does not eliminate the risk of disease development [137]. The duration of therapy in RHD patients depends on the severity of the disease, patient’s response and levels of inflammatory markers [136]. The relapse of inflammatory symptoms can occur after the cessation of treatment. In such cases, the re-introduction of treatment is required. It appears that only monthly benzathine benzylpenicillin G injections for five years (in patients without cardiac involvement) or 10 years (in those with cardiac involvement) from the last acute rheumatic fever episode or until the age of 21 years (whichever is longer) can effectively prevent the recurrence of ARF as well as the evolution to RHD in individuals who are at risk of this disease [138]. In patients experiencing adverse reactions related to beta-lactams, macrolide antibiotics, including azithromycin, erythromycin, clarithromycin and roxithromycin, are recommended [136]. The inciting streptococcal infection in patients with non-severe penicillin hypersensitivity can be treated with cefalexin and erythromycin for secondary prophylaxis. Azithromycin should be administered to patients with immediate penicillin hypersensitivity. However, frequently it is not so easy to implement and maintain such management. During the acute inflammatory stage of this disease, there are no therapies targeted at protection of cardiac valve damage. Rifaie et al. [36] demonstrated that the administration of colchicine (an anti-inflammatory drug) was associated with a decrease in CRP and IL-6 levels in RHD, which confirmed the presence of ongoing inflammation. One month treatment with colchicine, 0.5 mg twice daily, markedly decreased CRP (from 6.09 ± 4.39 (IU/mL) to 3.34 ± 3.07 (IU/mL)) and IL-6 (from 113.57 ± 37.41 (ng/L) to 45.57 ± 20.39 (ng/L) (*p* = 0.0001)). However, it is not confirmed whether such a reduction in inflammatory markers would translate into the delayed progression of the rheumatic process and valvular lesions. In vitro studies provided promising results concerning the use of hydroxychloroquine in RHD patients; however, clinical trial data are still not available [32,139]. In turn, corticosteroids are recommended in patients with severe carditis; however, they may not prevent subsequent rheumatic heart disease [13,137]. This type of treatment is effective in reducing the symptoms of acute rheumatic fever [140]. The results of one study demonstrated that L-carnitine helps to diminish myocardial injury in RHD patients undergoing cardiopulmonary bypasses [141]. L-carnitine was found to significantly reduce not only levels of myocardial injury markers (i.e., CK-MB, cTnI, hs-cTnT), but also hindered the activity of myeloperoxidase (MPO) and inflammatory cytokines in the myocardium. Moreover, it increased catalase (CAT) and superoxide dismutase (SOD) concentrations and limited the activation of activated nuclear factor erythroid 2-related factor 2 (Nrf2) and nuclear factor kappa B (NF-κB) [141]. Cardiovascular disease risk in rheumatoid disorders appears to be associated with autoimmune-mediated inflammation and subsequent development of atherosclerosis; thus, it was suggested that methotrexate or tumour necrosis factor-alpha (TNF-α) inhibitors could be used as treatment. However, such treatment was found not to decrease cardiovascular risk in this group of patients [142].

## 6. Conclusions

RHF remains an important cause of morbidity and mortality in developing countries. The results of studies mentioned in this review clearly show the involvement of the inflammatory state in the development and progression of this disease. The exact role of cytokines in inflammation sites remains to be examined, since most studies have, so far, focused on peripheral blood. Such analysis would provide information on inflammatory mechanisms in situ [6]. Medications with anti-inflammatory properties have been demonstrated to relieve symptoms; however, further research is required to answer the question of whether they will be able to prevent the development or progression of RHD.

## Figures and Tables

**Table 1:** The summary of studies concerning the role of inflammation and oxidative stress in RHD patients.

Population/Type of Material	Main Results	Ref.
Inflammation		
Mitral valve tissue (*n* = 28) from chronic RHD patients undergoing valve replacement surgery	The promotion of hypomethylation of various key inflammatory cytokines (TNFα, IL-6, and IL-8), integrin (ICAM1) associated with leukocyte transendothelial migration and extracellular matrix genes (vimentin, and laminin) observed in RHD.	[2]
Peripheral blood mononuclear cells from an Australian Aboriginal ARF	IL-1β amplifies expansion of GM-CSF cytokine axis in ARF peripheral blood mononuclear cells exposed to group A streptococcus. GM-CSF is a key T cell-derived effector cytokine in autoimmune diseases. CXCL10 may be involved in selective trafficking of GM-CSF to the heart. Hydroxychloroquine suppresses the expansion of GM-CSF-expressing CD4 T cells.	[32]
Formalin-fixed autopsy specimens from consecutive RHD patients	Inflammation may persist long after a bout of rheumatic fever leading to ECM remodelling. Significantly increased several phenotypes of DCs and increased substantial inflammatory cells such as macrophages and T lymphocytes in the LA of patients with RHD. Enlarged LA and AF in patients with RHD.	[29]
30 patients with rheumatic heart disease undergoing mitral valve replacement	Cardiomyocyte hypertrophy, nuclear enlargement, perinuclear clearing, sarcoplasmic vacuolation, fibrosis and inflammation in RHD patients with AF and NSR. Significant degenerative remodelling and ongoing inflammation associated with extensive fibrosis in RHD patients.	[47]
Human PBMCs isolated from heparinized blood of healthy donors	Human monocytes respond to bacterial RNA with secretion of IL-6, TNF and IFN-β (critically dependent on lysosomal maturation). TLR8 is the receptor for bacterial RNA in primary human monocyte-derived macrophages. TLR8-dependent detection of bacterial RNA is critical for triggering monocyte activation in response to infection with Streptococcus pyogenes.	[48]
Model of autoimmune heart inflammatory disease (myocarditis)	Innate GM-CSF appears critical for IL-6 and IL-23 responses by dendritic cells and generation of pathological Th17 cells in vivo. GM-CSF promotes autoimmunity by enhancing IL-6-dependent survival of antigen specific CD4(+) T cells.	[61]
13 valve specimens from nine patients with rheumatic carditis	Cellular infiltrates in AV and CAV primarily composed of T cells and macrophages. Majority of T cells in AV are of helper phenotype (Leu 3a). Expression of HLA-DR antigen by majority of the mononuclear cells and by vascular endothelium. Cells comprising the Aschoff body, primarily positive for the HLA-DR and a D8/17 (antigen present on the B cells of RF patients). Valvular injury appears at least partly mediated by delayed-type hypersensitivity mechanisms.	[62]
20 heart tissue infiltrates from 14 RHD patients	Predominance of IFN-gamma-, TNF-alpha- and IL-10-positive cells in RHD Predominance of IFN-gamma and TNF-alpha expression in the heart suggests that Th1-type cytokines could mediate RHD. Significantly lower IL-4 expression in valvular tissue may contribute to the progression of the RHD and permanent valvular damage (relative risk, 4.3; odds ratio, 15.8).	[63]
Patients with ARF and ARHD	Significant increase in CD4+ T cells, CD22+ B cells, and CD4:CD8 cell ratio and decrease in the percentages of CD8+ and CD3+ T lymphocytes in ARF and ARHD compared CRHD and normal controls. Higher proportion of IL-2R+ (CD25+) cells in PBMC in ARF and ARHD compared to CRHD or controls. Immunologically activated helper/inducer T cells may account for aberrations in lymphocytes distribution in peripheral blood of ARF and ARHD patients.	[65]
Surgical fragments obtained during valve correction surgery from 6 severe RHD patients	Inflammatory cytokines (IFN-gamma and TNF-alpha) are predominantly produced by heart-infiltrating T cells upon stimulation with LMM peptides. High percentage of reactivity against cardiac myosin reinforces its role as one of the major autoantigens involved in rheumatic heart lesions.	[66]
53 patients with ARF, 78 patients with chronic RHD vs. 20 normal control subjects and 39 patients with USP	Increased total leukocyte and lymphocyte counts in patients with RF and (to a lesser extent) in RHD compared with controls. Increased number of B cells and total T and T-helper-inducer (CD-4) cells in patients with ARF. Significantly higher total T and T-helper lymphocyte percentages and numbers in RF compared to RHD. Immunoregulatory defect in ARF: relative reduction of suppressor T cells, absolute increase in helper T cells and B cells leading to increased cellular and humoral immune response.	[67]
30 rheumatic mitral valves and in 15 control valves.	High TGF-beta 1 expression was observed in 70% of rheumatic mitral valves. Increased proliferation of myofibroblasts within rheumatic valves. Positive correlation between high TGF-beta1 expression and proliferation of myofibroblasts (*p* = 0.004), valvular fibrosis (*p* < 0.001), inflammatory cell infiltration (*p* = 0.004), neovascularization (*p* = 0.007) and calcification (*p* < 0.001) in the valvular leaflets. The ratio of proteoglycan to collagen deposition inversely correlated with TGF-beta 1 expression in mitral valves (*p* = 0.040).	[72]
89 with RHD	Higher levels of inflammatory cytokines in severe RHD compared with stable disease. Positive correlation between IL-6 and TNF-α expression in severe (but not in stable) RHD. IL-10 at baseline (HR 1.24, 95% CI 1.08–1.43, *p* = 0.003) and IL-4 (HR 1.12, 95% CI 1.01–1.24, *p* = 0.041) were predictors of events during the follow-up.	[75]
Cardiac tissue biopsies obtained from chronic RHD patients	Upregulated CCL3/MIP1α gene expression in myocardium. High expression of CCL1/I-309 and CXCL9/Mig in valvular tissue. Auto-reactive T cells infiltrating valvular lesions display a memory phenotype (CD4(+)CD45RO(+)) and migrate mainly toward CXCL9/Mig gradient. CXCL9/Mig is involved in mediating T cell recruitment to the site of inflammation in the heart.	[76]
27 patients with acute rheumatic fever (RF), 12 with only arthritis (RFA) and 15 with rheumatic heart disease (RHD)	Significantly increased TNF-α, IL-8 and IL-6 levels in the acute phase vs. after treatment. Higher increase in TNF-α and IL-8 levels in RHD patients with cardiac failure. Inflammatory cytokines, as TNF-α, IL-8 and IL-6 appear to play a pathogenic role in rheumatic fever.	[79]
Forty patients with rheumatic MVD and 23 controls	Significantly increased peripheral TH17 percentage and serum levels of TH17-related cytokine interleukin 17A, and decreased percentage of Treg cells in RMVD compared with controls. Significantly higher T helper 17/Treg ratio in RMVD patients compared with controls. Higher serum concentrations of hs-CRP in RMVD compared to control subjects. Increased serum levels of TGF-β1 in RMVD group compared with controls.	[84]
70 adults of RHD and 35 controls	Significantly lower level of Tregs (CD4+CD25(med-high)CD127(low) Foxp3(high)) in CD4+ T lymphocyte in RHD. patients compared to controls (median 0.6% versus 3.2%; *p* = 0.001) Significantly lower Treg count in patients with multivalvular-disease only.	[85]
Cardiac tissues of 11 RHD patients and 11 controls	Significantly down-regulated miR-101 in cardiac tissue of RHD patients (*p* = 0.011). TLR2 is a direct target gene of miR-101. miR-101 knock-down is related to over-stimulated immune response in PGN-activated THP-1 cells. Significantly higher concentration of TNF-α (*p* = 0.0017), IL-1β (*p* = 0.015) and IL-6 (*p* = 0.014) in serum samples of RHD patients.	[87]
Rheumatic heart disease (RHD) patients and healthy controls	133 miRNAs (i.e., miR-1183 and miR-1299) significantly upregulated in RHD patients compared with controls. 137 miRNAs (i.e., miR-4423-3p and miR-218-1-3p) significantly downregulated in RHD patients. miR-1183 and miR-1299 appear to play distinct roles in RHD pathogenesis accompanied by secondary PAH.	[88]
80 patients with rheumatic MS: group 1—35 patients with rheumatic mitral stenosis and left atrium; group 2—45 patients with rheumatic mitral stenosis without left atrium	Significantly elevated levels of plasma MDA, protein carbonyl and total oxidant status in gr.2 vs. gr. 1 Significantly decreased total antioxidant status levels in gr.1 vs. gr. 2. Significantly increased hs-CRP, TSA and PBSA levels in gr. 1 vs. gr 2.	[105]
314 patients with RMVS, 57 healthy persons in control group	Significantly higher NLR in patients with RMVS (2.9 (0.6–13.0) vs. 2.1 (0.7–5.8), *p* < 0.001). Higher C-reactive protein in the RMVS group (5.99 (0.3–23.7) vs. 2.98 (0.6–6.3), *p* = 0.001). NLR (OR: 2.24, *p* = 0.04), CRP (OR: 1.34, *p* = 0.03) and left atrial diameter (OR: 1.21, *p* = 0.001), independent predictors of RMVS.	[108]
Oxidative stress		
56 patients who underwent valve replacement due to rheumatic (*n* = 32) and degenerative (*n* = 24) heart valve disease.	Significantly higher incidence of mitral stenosis in patients with rheumatic HVD vs. degenerative HVD (*p* < 0.05). Significantly lower tissue TAC in patients with rheumatic HVD (*p* = 0.027) vs. degenerative HVD. Similar TOS and OSI in two HVD groups (*p* > 0.05).	[122]
25 rheumatic HVD paediatric patients and 25 paediatric congenital HVD patients and 20 healthy age-matched control subjects	Significantly higher serum TnC level of the patients with rheumatic HVD (median 9.09 (0.94–46.30) ng/mL) compared with congenital HVD and control groups (median 2.97 (0.66–11.80) ng/mL; *p* < 0.01, 4.72 ± 1.77 ng/mL; *p* < 0.05, respectively). Similar levels of serum TAC, TOS and OSI in all groups. No correlations between the level of TnC, TOS and OSI.	[127]
90 patients with CRVD	Significantly elevated levels of AOPP and hs-CRP in CRVD patients compared to controls (AOPP 212.62 +/− 34.14 μmol/L vs. 126.97 +/− 27.74 μmol/L *p* < 0.00006 and for hs-CRP 5.40 +/− 1.98 mg/L vs. 2.66 +/− 1.36 mg/L *p* < 0.05). Association between AOPP and the presence of prosthetic valve and time after surgery (*p* < 0.0008 and *p* < 0.005, respectively). No correlation between the levels of AOPP and hs-CRP with age, sex and degree of mitral valve stenosis.	[128]

## Data Availability

Not applicable.
